# Contrasting energy pathways at the community level as a consequence of regime shifts

**DOI:** 10.1007/s00442-013-2878-2

**Published:** 2014-01-12

**Authors:** Jun Xu, Zhourui Wen, Zhixin Ke, Meng Zhang, Min Zhang, Nichun Guo, Lars-Anders Hansson, Ping Xie

**Affiliations:** 1 Donghu Experimental Station of Lake Ecosystems, State Key Laboratory of Freshwater Ecology and Biotechnology of China, Institute of Hydrobiology, Chinese Academy of Sciences, Wuhan, 430072 People’s Republic of China; 2 Hubei Fishery Science Institute, Wuhan, 430071 People’s Republic of China; 3 Key Laboratory of Marine Bio-resource Sustainable Utilization, South China Sea Institute of Oceanology, Chinese Academy of Sciences, Guangzhou, 510301 People’s Republic of China; 4 Jiangxi Academy of Environmental Sciences, Nanchang, 330029 People’s Republic of China; 5 College of Fisheries, Huazhong Agricultural University, Wuhan, 430070 People’s Republic of China; 6 Resources and Environment College, Anhui Agricultural University, Hefei, 230036 People’s Republic of China; 7 Institute of Biology/Aquatic Ecology, Ecology Building, Lund University, Lund, 22362 Sweden

**Keywords:** Energy pathways, Food web, Omnivory, Stable isotopes, Niche space

## Abstract

Ecological regime shifts typically result in abrupt changes in ecosystem structure through several trophic levels, which leads to rapid ecosystem reconfiguration between regimes. An interesting aspect of the impact of regime shift is that alternative regimes may induce distinct shifts in energy pathways; these have been less tested than structural changes. This paper addresses this by using stable isotopes to establish the energy pathways in fish communities. We specifically focus on the impact of regime shift on changes of the energy pathways, and how the magnitude and direction of these changes affect the local community. We found that energy pathways significantly varied among the planktivorous, benthivorous, and piscivorous trophic guilds as a result of the alternative regimes. The regime shift from a clear to a turbid state altered the food web towards planktonic energy pathways and truncated food chain length, which is indicative of less ecological efficiency. This was confirmed by the adaptive foraging strategies of prevalent omnivores in the current communities. These structural and functional characteristics of trophic interactions might not facilitate classic trophic cascading effects in such a turbid regime and suppress the system’s response to environmental changes, e.g., nutrient loading, and restoration efforts in turbid to clear water regime shifts.

**Electronic supplementary material:**

The online version of this article (doi:10.1007/s00442-013-2878-2) contains supplementary material, which is available to authorized users.

## Introduction

Ecosystems that undergo rapid regime shifts are characterized by a transformation in trophic structure and dynamics (Scheffer and Carpenter 2003). In aquatic ecosystems, the best-described regime shifts are distinguished by different primary producers. Shallow marine systems can shift between coral- and macroalgae-dominated structures and grazers (McCook 1999), and shallow lakes can shift between clear, macrophyte-dominated and turbid, phytoplankton-dominated regimes (Scheffer et al. 1993). Understanding variations in these alternative regimes is of importance for understanding responses to environmental changes and ecosystem management. For instance, biomanipulation of the fish community has been used to overcome biological resistance following nutrient loading reduction (Hansson et al. 1998; Jeppesen et al. 2007). When zooplanktivorous fish are removed from shallow lakes, the resulting release from predation allows zooplankton to control the phytoplankton biomass. The improved light condition, in turn, facilitates the establishment of macrophytes (Jeppesen et al. 1990).

Ecological regime shifts typically result in abrupt changes in ecosystem structure through several trophic levels, which leads to rapid ecosystem reconfiguration between regimes (Andersen et al. 2009). The stability of alternative regimes is also achieved by a number of different mechanisms. For instance, the presence of macrophytes is a structural change that can maintain the clear water regime through allelopathy and resource competition (Scheffer et al. 1993), provide predation refuges for zooplankton, and reduce sediment suspension (Jeppesen et al. 2007). An increase in resources and habitat structures in a clear water regime consequently results in a higher biodiversity of invertebrates and higher trophic level consumers (Brönmark et al. 2010; Scheffer and Carpenter 2003). In turbid regimes primary production is dominated by phytoplankton, whereas macrophytes are restricted to a narrow littoral zone. The stability of the turbid regime is achieved by other mechanisms, for example facilitated recycling of nutrients by benthic fishes (Jeppesen et al. 2007), or nutrient release by fish feeding directly on macrophytes (Hansson et al. 1987); processes which further promote excessive phytoplankton development.

In addition, regime shifts will also affect lake ecosystems, not only structurally but also functionally. An interesting aspect is that the alternative regimes may induce distinct shifts in the energy pathways. For instance, changes in primary production associated with regime shifts have a large bottom-up effect from primary consumers to the fish community, which can lead to wide-scale re-functioning of the whole food web (Hansson et al. 2010; Hargeby et al. 2004). However, an important question is how a current regime may alter the energy pathways. For instance, eutrophication, which is induced by nutrient loading, is a typical problem of the pelagic zone and drives the primary production towards phytoplankton dominance (Jeppesen et al. 2007). This results in light limitation of benthic primary producers and further reduction of the amount and distribution of benthic primary production in the whole system (Jeppesen et al. 2007; Scheffer and Carpenter 2003). Despite the general pattern of benthic and planktonic energy pathways, our knowledge about the effects of energy pathways in alternative regimes on fish communities remains negligible, especially for eutrophic subtropical shallow lakes dominated by omnivorous fish species. With respect to such subtropical lakes, the following questions remain unanswered:

1. Do fish communities in turbid water regimes have different energy pathways compared to those in clear water regimes?
2. Are fish species of diverse foraging niches (generalist vs. specialist) affected by different magnitudes of food web properties, i.e., benthivory and trophic positions?

One of the more well-studied sub-tropical flood plain lakes is Lake Taihu, China, which has undergone massive eutrophication in the last four decades. This lake is therefore an ideal ecosystem for examining changes in alternative regimes (Guo 2007; Qin et al. 2007). Here we addressed the benthic and planktonic energy pathways of the lake by using stable isotopes, and focused on the direction and magnitude of changes in both trophic position and energy pathways of the fish communities across clear water and turbid regimes in space and time. We also incorporated dietary data of fish species by conducting a comprehensive literature search to identify the foraging strategies and trophic niche diversity of fishes within these regimes. We illustrate the manner in which alternative ecosystem regimes can affect the energy pathways of fish communities, giving insight into the causes and patterns, as well as fundamental stability properties, of this ecosystem.

## Materials and methods

### Sampling areas

Lake Taihu is located in the Yangzi River delta in the East Plain of China. It has a maximum depth of 2.6 m, a mean depth of 1.9 m, and a surface area of 2,338 km^2^ (Qin et al. 2007). The lake is of great importance for water supply, flood control, agriculture, shipping, and recreation (Guo 2007; Qin et al. 2010). During the past decades, the lake has undergone a steady increase in eutrophication with a regular occurrence of cyanobacterial surface blooms in the warm seasons of each year (Guo 2007; Qin et al. 2007). Meiliang Bay (MB), with a water surface area of 125 km^2^, accommodates municipal and industry wastewater from Wuxi City and is the most eutrophic part of the lake with extremely dense accumulation of *Microcystis* blooms during the warm season. Gonghu Bay (GB), located at the northeast portion of Taihu Lake, presented abundant submerged plants during our first sampling period but underwent a rapid regime shift afterwards, resulting in a dramatic change in the local light and nutrient environment due to a hydrological and/or nutritional regime shift (Hu et al. 2008; Qin et al. 2010). Both bays have flattened bottom topography (depth range 1.8–2.0 m) and high habitat homogeneity with no significant difference in space of the stable isotope compositions of the benthic primary consumers used here.

### Sampling and pretreatment

Samples of water for physiochemical parameters from the clear and turbid water regime surrogates were tested in three sites in each of the bays (Fig. S1). Sampling was conducted once monthly during the growth season (May–October; *n* = 6) in 2005 and 2008, respectively. Water transparency was measured in situ with a Secchi disc. Water samples were collected at each site with a Tygon water sampler for chemical analysis of nutrients, and then kept in 1-L acid-cleaned polyethylene bottles that were transported to the laboratory. Nutrient concentrations were then determined using standard techniques (Eaton and Franson 2005). Based on these results (Table S1), we classified the ecosystem regimes as the turbid water regime in Meiliang, 2005 (Turbid-M), the turbid water regime in Gonghu Bay revisited, 2008 (Turbid-G), and the clear water regime in Gonghu Bay, 2005 (Clear-G). Under the assumption that the ‘clear water’ bay was a good reference site for comparison with the ‘turbid’ bays, the three bays were analyzed as three independent systems in this study.

Samples of fishes and baseline macroinvertebrates were conducted at Meiliang Bay and Gonghu Bay during the first week of October 2005, and revisited at Gonghu Bay during the first week of October 2008. Thus, the sampling occurred at the same time period (i.e., the end of the growing season) in each sampled year between locations (GB, MB) or at each sampled location between years (GB). This procedure was assumed to control the intra-annual cycles in the stable isotope values of primary consumers. Based on local observations of fishery catches, selected fishes and benthic macroinvertebrates for analysis co-occurred and were dominant in these areas.

Fishes were collected by volunteer fishermen from four random transects from littoral to pelagic areas in each bay. These species were selected because their biomass accounted for more than 85 % of total fishery catches and they are persistent to and dominant in both clear and turbid water regimes in the subtropical flood plain lakes (Zhang et al. 2013). We collected tissue samples from six individuals each of up to six commonly occurring larger fish species (generally ≥200 mm in length) from each random transect within the areas mentioned above: redfin culter (*Culter erythropterus*), common carp (*Cyprinus carpio*), goldfish (*Carassius auratus*), silver carp (*Hypophthalmichthys molitrix*), bighead carp (*Aristichthys nobilis*), and lake anchovy (*Coilia ectenes taihuensis*); this reduces possible bias due to the collection of small individuals that are undergoing a foraging shift in their early life cycles. To avoid intraspecies bias for smaller fishes (generally ≤100 mm in length), we collected tissue samples from 15 individuals each of up to two commonly occurring fish species from each random transect: icefish (*Neosalanx taihuensis*) and freshwater garfish (*Hyporhamphus intermedius*). The sacrificed fishes in fishery catches were placed on ice and their white dorsal muscle tissue was dissected in the laboratory.

Benthic macroinvertebrates (mussels and snails) for stable isotope analysis were collected by the volunteer fishermen along each transect using hand-held nets and trawl nets. Fifteen large individuals (shell height >15 mm) of grazing snail, *Bellamya aeruginosa*, and filter-feeding mussels, *Cristaria plicata* and *Anodonta woodiana woodiana*, were collected per transect as benthic and planktonic baseline indicators, respectively, to reduce individual-level variation of isotopic composition based on a pilot isotope baseline study (Xu et al. 2011). Mussels and snails were placed on ice and a sample of foot muscle tissue was taken in the laboratory for stable isotope analysis.

Mussel tissues of animals were dried in a drying oven at 60 °C to a constant weight. Samples were then ground to a fine homogeneous powder with a mortar and pestle. The mortar and pestle was acid-washed and dried to prevent cross-contamination between samples. Samples were put into tin capsules for stable isotope analysis.

### Literature survey of dietary data

Dietary data were collected by conducting a comprehensive literature search, followed by examination of the citations included in the original studies. All data came from the lakes located in the middle and lower area of the Yangtze River, where the current studied lake, Lake Taihu, is located. Some published studies divided diet data into groups based on year, season, month, and size of fish. These different diet data were averaged for each lake (Zhang et al. 2013). The data of multiple studies from the same ecosystem were also averaged. To be comparable with our stable isotope analysis, we only kept diet data for adult fish for analysis when adult and juvenile fish were implicitly reported. Although the degree of taxonomic detail of prey items in the previous studies were highly variable, it was usually possible to classify the prey organisms into the following groups: planktonic primary producers, zooplankton, benthic primary producers, zoobenthos and fish prey. We deleted prey categories reported as: ‘unknown,’ ‘other,’ or ‘miscellaneous,’ but kept the category sum to 100 % (Zhang et al. 2013). Trophic guilds of fishes were thus determined according to their dominant prey item, including benthivores (the benthivorous fishes *H. intermedius, C. carpio* and *C. auratus*), piscivores (the piscivorous fishes *C. erythropterus* and large *C. ectenes*), and planktivores (the planktivorous fishes *N. taihuensis*, *H. molitrix* and *A. nobilis*).

### Stable isotope analysis

The stable C (δ^13^C) and N isotope ratio (δ^15^N) values were generated after analysis of samples on a Delta Plus (Finnigan, Bremen, Germany) continuous-flow isotope ratio mass spectrometer coupled to a Carlo Erba NA2500 elemental analyzer (Carlo Erba Reagenti, Milan, Italy). Stable isotope ratios were expressed in δ notation as parts per thousand (‰) deviation from the international standards according to the equation: δ*X* = [(*R*_sample_/*R*_standard_) − 1] × 1,000, where *X* is ^15^N or ^13^C and R is the corresponding ratio ^15^N/^14^N or ^13^C/^12^C. δ is the measure of heavy to light isotope in the sample, whereby higher δ values denote a greater proportion of the heavy isotope. The standard for N was atmospheric N and that for C was Vienna Pee Dee belemnite. The reference material for δ^15^N was ammonium sulfate (IAEA-USGS25), and that for δ^13^C was carbonate (IAEA–NBS18), supplied by the US Geological Survey (Denver, Colombia) and certified by the International Atomic Energy Agency (Vienna, Austria). On a daily basis, an internal working standard, urea (δ^15^N = −1.53 ‰, δ^13^C = −49.44 ‰), was employed. Twenty percent of the samples were run in duplicate; the average SEs of replicate measurements for δ^13^C and δ^15^N were both <0.3 ‰.

### Benthivory and trophic position calculations

To convert δ^13^C and δ^15^N to more ecologically meaningful metrics, namely benthivory (an indicator for benthic energy pathways) and trophic position (Vander Zanden et al. 2003), we incorporated baseline adjustments to eliminate the effects of differing isotopic composition in different regimes. We estimated benthivory, defined as the estimated reliance on littoral benthic resources, of each individual fish using δ^13^C data and a two-source mixing model (Vander Zanden and Rasmussen 2001): benthivory = (δ^13^C_fish_ − δ^13^C_mussel_)/(δ^13^C_snail_ − δ^13^C_mussel_). Littoral grazing snails (*B. aeruginosa*) and filter-feeding mussels (*C. plicata* and *A. woodiana woodiana*) were used as benthic and planktonic end members, respectively, for each transect. We assumed that there is no trophic enrichment in δ^13^C (trophic fractionation = 0 ‰), since estimates of C fractionation broadly overlap with zero (Post 2002), and using a different value does not change the overall conclusions, only the actual benthivory estimates.

To capture potential spatial heterogeneity in δ^15^N baselines for fishes that feed on both planktonic and benthic food webs, trophic position was calculated as trophic position = δ^15^N_fish_ − [δ^15^N_mussel_ × (1 − benthivory) + δ^15^N_snail_ × benthivory]/3.4 + 2.0, where the contribution of mussel and snail δ^15^N was weighted by the estimated benthivory value for each individual fish species. Mussels and snails were assumed to have a trophic position of 2.0. Several recent studies have synthesized trophic level fractionation of δ^15^N from the literature. Here, we used a universal trophic fractionation value of 3.4 ‰ to ensure consistency in methodology with previous comparative food web studies (Post 2002; Vander Zanden and Rasmussen 2001). Note that these models use primary consumers (rather than primary producers) as isotopic baselines and thus estimate the contributions of pelagic and benthic secondary production, assuming that the mixing is linear (Post 2002). Stable isotopic values of baseline organisms in each transect were used for the calculation of benthivory and trophic position of the fishes sampled from each transect in each regime.

### Statistical analysis

Overall, δ^13^C vs. δ^15^N bi-plots of each fish species in each regime with standard ellipse areas (SEA) were used to compare baselines and fish species among regimes. SEA was estimated using multivariate ellipse-based metrics, which are bivariate equivalents to SDs in univariate analysis, and contains ca. 40 % of the data regardless of sample size (Jackson et al. 2011). We used a Welch two-sample *t*-test after testing for homogeneity of variance for comparisons of δ^13^C (and δ^15^N) of long-term primary consumers, mussel and snail, to test the utility of stable isotope analysis for determining the energy baselines of benthic and planktonic food webs in each regime. If δ^13^C and δ^15^N of mussel and snail are not distinct from each other, the following circular statistics and mixing models are not appropriate. Significance levels were set at *α* = 0.05 throughout.

We used circular statistics to quantify directional shifts in stable isotope space (δ^13^C − δ^15^N) for fish communities in time and space (Schmidt et al. 2007). Circular statistics is an area of mathematics concerned with the analysis of angles ranging from 0° to 360° (Batschelet 1981). Because shifting of the baseline stable isotope signatures can be transferred through food chains, we corrected δ^15^N values of all fish species (δ^15^N_c_ = δ^15^N_fish_ − δ^15^N_baseline_) before circular statistics (Schmidt et al. 2007). Significant isotopic shifts in time or space are related to hypotheses regarding planktonic and benthic resource contributions in alternative regimes. We measured the direction (angle) and magnitude (arrow length) of changes in fish averages in stable isotope space between the clear water regime (Clear-G) and the turbid water regimes (Turbid-G and Turbid-M). We fit a von Mises distribution (known as the ‘circular normal distribution’) to our sample of angles and produced maximum likelihood estimates of the corresponding population parameters (Batschelet 1981). For community level and trophic guild comparisons, we calculated mean vectors including the mean angle (*μ*) and the concentration of angles (*r*), derived from magnitudes and directions of fish species vectors in stable isotope space (Batschelet 1981). In order to visualize the direction and magnitude of community shifts, we used arrow diagrams to plot the angle of change and the mean vector used for the between-regime comparisons. In order to quantify regime-shift effects, we used Rayleigh’s test for circular uniformity to determine whether the sample of angles of community level and trophic guilds significantly differed from random indicating directedness for the between-regime comparisons (Batschelet 1981).

Watson–William’s test was used to compare trophic guilds of fishes between regimes to find whether movements of trophic guilds in stable isotope space were significantly different from one another. All circular graphs, descriptive statistics, and statistical tests were performed using Oriana 4.00 (Kovach Computing Services, Anglesey, UK).

We tested the null hypothesis of no differences in energy pathways of fish communities among regimes using one-way ANOVA followed by Tukey’s post hoc test, with regimes (three levels: Turbid-M, Turbid-G, and Clear-G), trophic guilds (three levels: benthivores, piscivores and planktivores) and fish taxa (eight fish species) as fixed factors and benthivory and trophic position as response variables (*n* = 4 transects).

For the dietary data, we compared the percentage of ingestion of different food resources for fish species from turbid and clear water regimes in lakes. We first transferred diet data matrices to distance matrix based on the Bray-Curtis index (Bloom 1981) and used the Mantel statistic for the similarity of two matrices based on Pearson’s product-moment correlation with 999 permutations. A difference in the ingestion percentage of these prey categories would indicate that habitat and prey use differed across fish taxa between turbid and clear water regimes.

## Results

Nutrients (total N and P) and chlorophyll *a* (used as a proxy for algal biomass) were significantly higher (1.1- to 2.4-fold) during turbid (Turbid-M and Turbid-G) than clear water regimes (Clear-G, *P* < 0.01 except for total N; Table S1). Moreover, the clarity (as indicated by Secchi depth) and the coverage of submersed macrophyte were significantly higher (more than threefold) in the clear water regime that those in turbid water regimes (*P* < 0.005 for all comparisons).

**Fig. 1:**
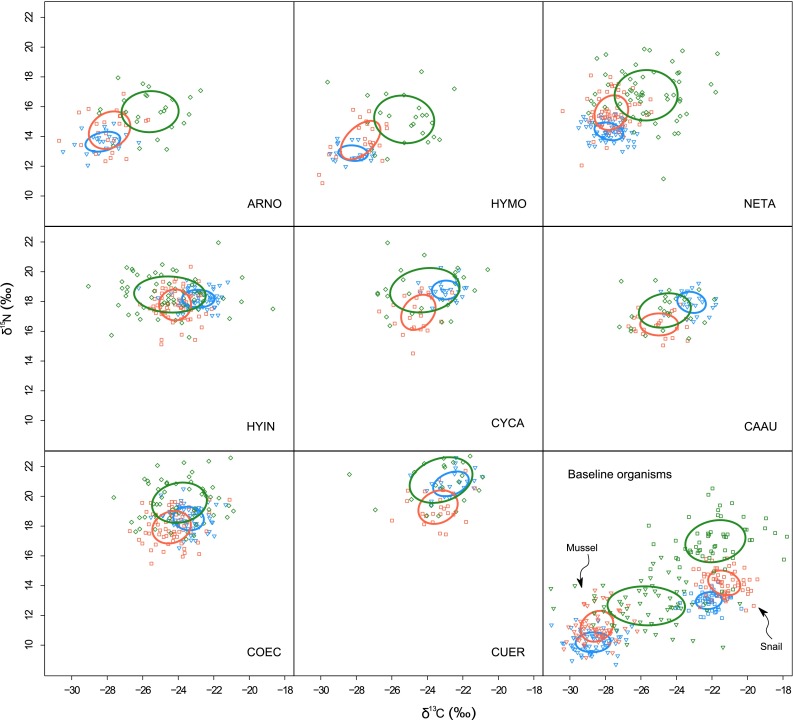
Stable C (δ^13^C) and N isotope ratio (δ^15^N) bi-plots for fishes, mussels and snails under each regime illustrating the isotopic niche of these species in different regimes. *Blue symbols* represent individual organisms in the clear water regime (Clear-G), *red symbols* represent individual organisms in the turbid regime of Gonghu Bay (Turbid-G), and *green symbols* represent individual organisms in the turbid regime of Meiliang Bay (Turbid-M).* Lines* enclose the standard ellipse area for each species (*n* = 18–45) from each regime. *GUER* *Culter erythropterus*, *COEC* *Coilia ectenes*, *NETA* *Neosalanx taihuensis*, *HYIN* *Hyporhamphus intermedius*, *CYCA* *Cyprinus carpio*, *CAAU* *Carassius auratus*, *HYMO* *Hypophthalmichthys molitrix*, *ARNO* *Aristichthys nobilis*

In each regime, δ^13^C and δ^15^N of snails and mussels were distinct (*P* < 0.001; Fig. 1; Table S2); therefore circular statistics and mixing models were applied to fish communities in different regimes. Mussels were ^13^C and ^15^N depleted compared to snails, allowing for the discrimination between base resources of food webs. Differences in δ^13^C and δ^15^N of fish species by regimes were complex, and it was difficult to identify patterns of food web change from these isotopic signatures. However, some species, such as those belonging to the planktivorous trophic guild, had clearly shifted in isotopic niche space by regimes (Fig. 1). This is likely because the δ^13^C and δ^15^N of consumers were affected by both a biogeochemical shift of habitats and a foraging strategy shift of consumers in these areas (Xu et al. 2007c).

**Fig. 2a, b:**
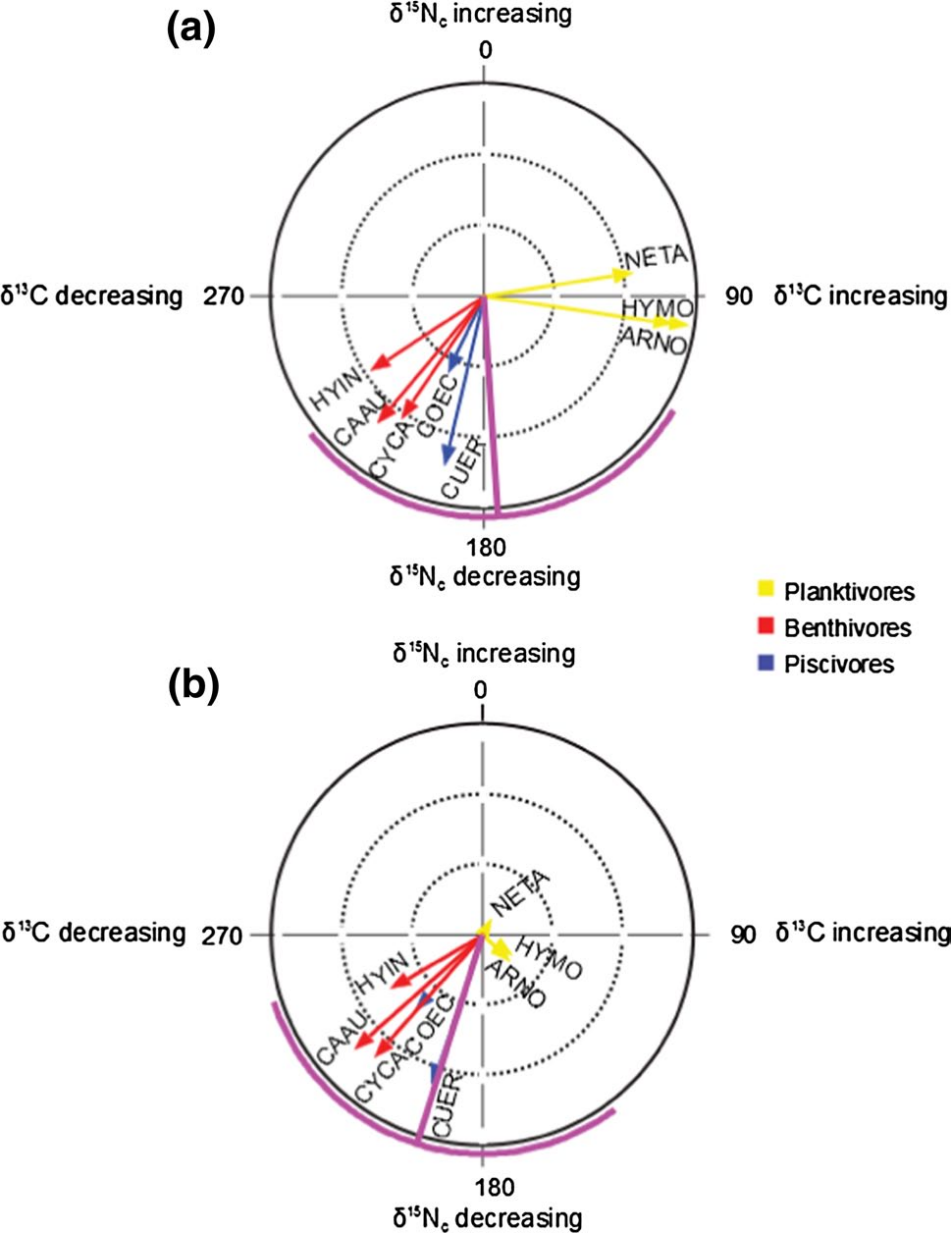
Circular arrow diagrams for direction and magnitude for fish species impacted by alternative regimes. **a** Directional food web differences between clear and turbid water regimes in space and **b** in time. Each *arrow* represents the direction of shift in isotopic niche space among regimes for a species. The* length* of the *arrow* corresponds to the magnitude of change for that species. Each *color* represents a trophic guild. Trophic guilds of fishes were determined according to their dominant prey item, including the planktivorous fishes, *N. taihuensis*, *H. molitrix* and *A. nobilis*, the benthivorous fishes, *H. intermedius, C. carpio* and *C. auratus*, and the piscivorous fishes, *C. erythropterus* and large *C. ectenes*. The *straight pink line* is the mean vector of change among all species; the* curved pink line* indicates the 95 % confidence interval around the mean vector of change. Note that δ^15^N_c_ on the *y*-axis was the δ^15^N value corrected by isotopic baselines and thus indicates trophic position of the shift

We found considerable directional difference between clear and turbid regimes as indicated by the circular statistic (Fig. 2). Community-wide shifts in stable isotope space exhibited no significant directionality (Table 1), but the results varied among the trophic guilds. In terms of shift in time (Clear-G vs. Turbid-GR), Benthivores showed significant shifts in direction between clear water and turbid regimes. Significant shifts in space also occurred for benthivores and planktivores. Further, comparison of directional shifts between trophic guilds showed that benthivores and piscivores exhibited similar directional changes, which were marginally significantly different from the directional change of planktivores between clear and turbid regimes (Table 1).

**Table 1:** Directional statistics quantifying change in isotope niche space for community level and trophic guilds between clear water and turbid regimes in time and space

	No. observations	Mean vector		Circular SD	Rayleigh’s test		Watson–William’s test
		*μ*	*r*		*Z*	*P*	
Clear to turbid water regime in time (Clear-G to Turbid-G)							
Community	8	197.47	0.52	65.30	2.182	0.111	
Planktivores	3	100.81	0.69	49.83	1.408	0.266	a
Benthivores	3	229.96	0.99	7.64	2.947	*0.036*	b
Piscivores	2	210.58	0.98	11.54	1.920	0.153	b
Clear to turbid water regime in space (Clear-G to Turbid-M)							
Community	8	176.34	0.52	65.65	2.152	0.115	
Planktivores	3	92.46	0.99	8.11	2.940	*0.037*	a
Benthivores	3	223.75	0.99	9.70	2.915	*0.038*	b
Piscivores	2	199.30	0.99	6.05	1.978	0.141	ab

Directional differences between trophic guilds were tested with Watson–William’s test and the* same letters* indicate no significant difference at *P* < 0.05

*Clear-G* Clear water regime; *Turbid-G* turbid regime of Gonghu Bay;* Turbid-M* turbid regime of Meiliang Bay; *μ* mean angle; *r* concentration of angles, derived from magnitude and direction metrics of each fish species

*Italic* indicates significant difference at *P* < 0.05

**Fig. 3:**
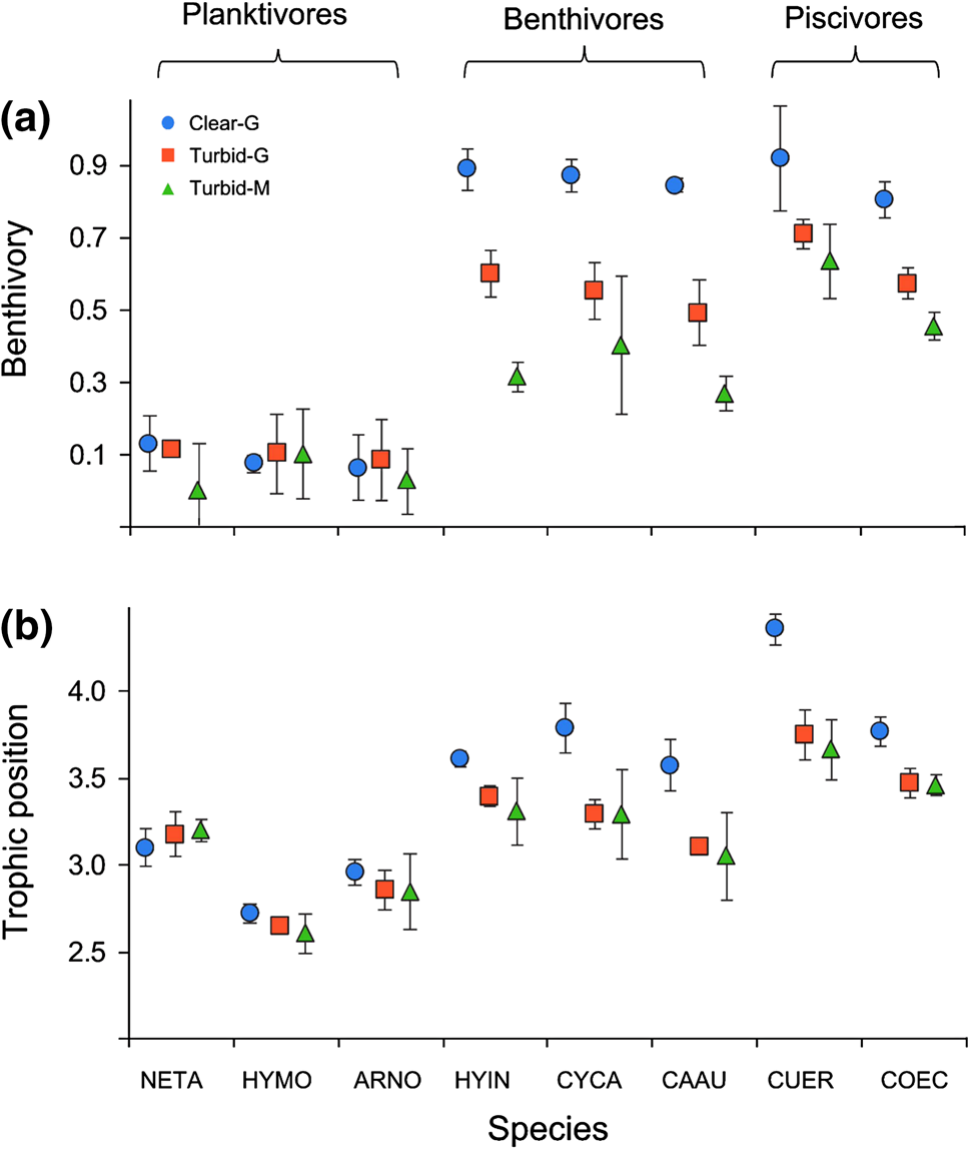
Benthivory (**a**) and trophic position (**b**) of fish species in clear water and turbid regimes (*n* = 4 transects). *Blue circles* Clear G, *red squares* Turbid G, *green triangles* Turbid M.* Error bars* represent SDs. Abbreviations as in Fig. 1

**Fig. 4:**
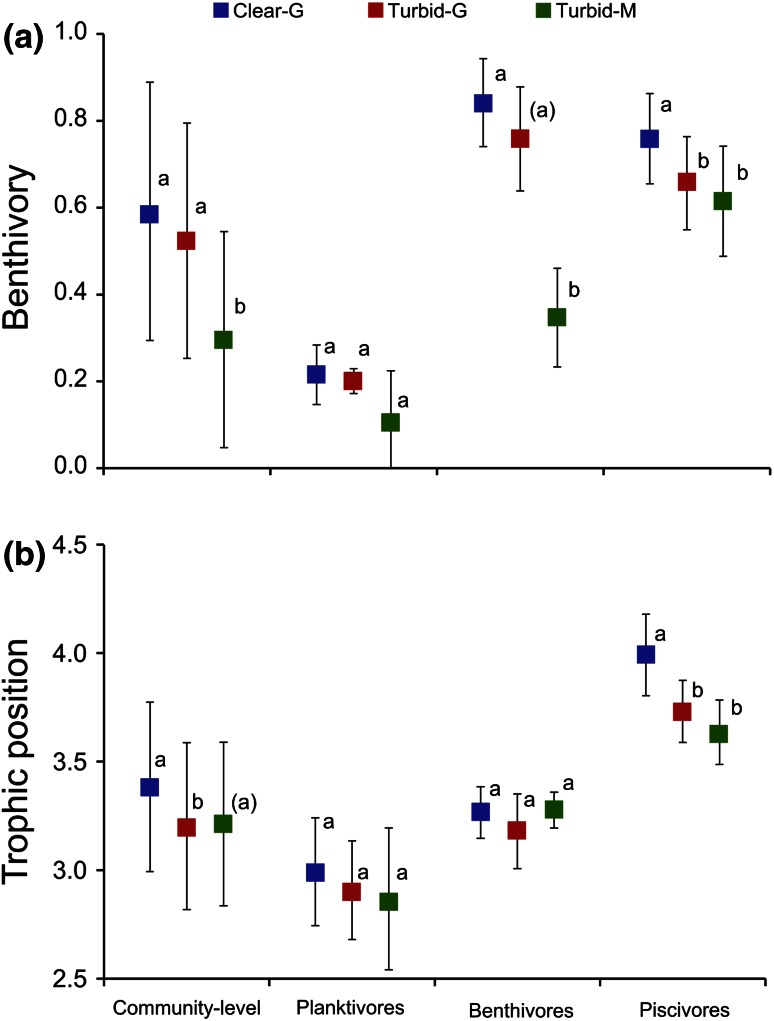
Differences in benthivory (**a**) and trophic position (**b**) of fishes at community (*n* = 32) and trophic guild levels among regimes (*n* = 8–12). *Blue squares* Clear G, *red squares* Turbid G, *green squares* Turbid M.* Different letters* indicate significant difference at *P* < 0.05;* letters in parentheses* indicate a marginal significant difference at 0.06 < *P* < 0.05. *Error bars* represent SDs. Abbreviations as in Fig. 1

Fish benthivory and trophic position varied substantially among species (Fig. 3). There were significant differences in mean benthivory and trophic position in the effects of alternative regimes at the community level (Fig. 4). Significant differences in benthivory were found in the trophic guilds of benthivores and piscivores, though not in planktivores, indicative of a lesser response of planktivory to regime shifts. Benthivores and planktivores did not show a significant difference in trophic position among regimes, while the trophic position of piscivores was significantly lower in the turbid regimes (Fig. 4).

**Fig. 5:**
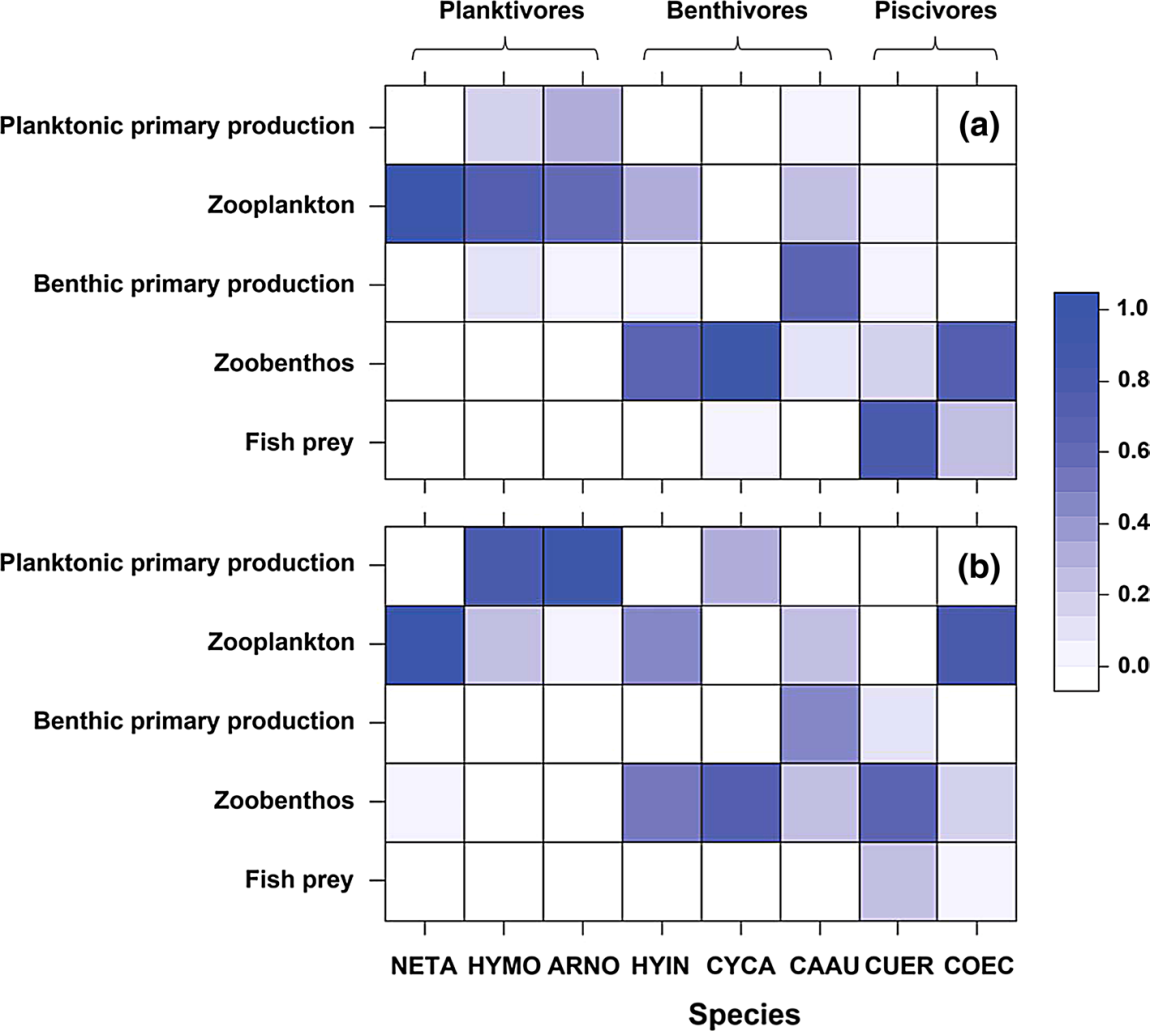
Ingestion of different food sources by fish species in clear (**a**) and turbid (**b**) water regimes indicating the foraging strategy shift. Abbreviations as in Fig. 1

We tested the hypothesis that diet compositions of fish communities (Tables S3, S4) were similar to each other in the clear and turbid regimes. We found no similarity between the composition of food sources ingested by these fish species in turbid and clear water regimes (*r* = 0.2334, *P* = 0.126). The general decrease in fish prey of piscivorous fishes and decrease in benthic food sources of both piscivorous and benthivorous fishes were observed to be related to alternative regimes of clear and turbid water (Fig. 5), supporting the mixing-model results.

## Discussion

Shallow lakes are the typical example of ecosystems with alternative regimes. In eutrophic shallow lakes, the regimes are commonly categorized as the clear water regime with abundant submersed macrophytes and the turbid regime with few submersed macrophytes (Jeppesen et al. 2007; Scheffer and Carpenter 2003). The regime shifts from macrophyte to phytoplankton dominance are often associated with a loss of habitat heterogeneity. For example, submersed plants create buffers to the effects of increasing external nutrient loading and thus provide a gradient of nutrient in local habitats (Scheffer and Carpenter 2003). Meanwhile, the provision of refuges for prey animals by macrophytes reinforces the stability and persistence of the relatively complex local food web interactions during clear water regimes (Jeppesen et al. 2007). These mechanisms seem to be weaker in the turbid regime because of the dominance of planktonic algae primary production. For example, as found in this study, the benthivore and piscivore fish species had a regime-shifting effect, reducing energy sources from the benthic food web and increasing planktonic energy pathways, due to the reduction in habitat heterogeneity and the enhancement of phytoplankton productivity. Therefore, the distribution and availability of resources with respect to regime shift changed both structure and energy pathways of the local food web in this lake.

### The baseline isotopic shifts between alternative regimes

Long-lived primary consumers, such as mussels and snails, were found to be good temporal integrators of the isotopic variation at the base of the food webs, which effectively reflected spatial differences between different food webs. The isotopic signature of snails approximates that of periphyton and detritus that form the base of the littoral or benthic food web, while the isotopic compositions of mussels is similar to that of the seston that forms the base of the pelagic or planktonic food web (Cabana and Rasmussen 1996). As indicated by our stable isotope results of primary consumers, the turbid water regime generally gave a higher and more variable isotopic signal than the clear water regime. This can be attributed to the enrichment of stable isotopes and variations in primary productivity during the turbid regime (Gu et al. 2006; Xu et al. 2007c). Another important reason for enriched ^15^N is the input of wastewater with high NO_3_^−^–δ^15^N values from the watershed (Cabana and Rasmussen 1996). The food web in the turbid water regime of Meiliang Bay is an example of anthropogenic contamination because this system has had integrated sewage inputs over long time periods and consumer ^15^N reflects the movement of waste through the food chain compared with the clear water regime in Gonghu Bay (Wen et al. 2010; Xu et al. 2007a).

The isotopic shift observed for snails and mussels in alternative regimes could also result from the change in the contribution of planktonic and benthic C sources. For example, *B. aeruginosa* consumed a wide range of food items, including benthic organic detritus, sand grains, and algae (including cyanobacteria, diatoms and green algae) (Xu et al. 2007b). Although algae typically accounted for <10 % of the total diet composition, there is evidence that *B. aeruginosa* increases the intake of planktonic resources with the increase of planktonic production (Fang et al. 2012). Mussels in this study (*A. woodiana* and *C. plicata*) fed primarily on algae, but detritus has previously been recorded, to some extent, in the total diet composition (Xu et al. 2007b). Studies on other species of bivalves indicate that they selectively ingest particulate organic matter, such as phytoplankton, microbenthos, detritus, and sedimentary organic matter from the water column and/or surface sediment (Garton et al. 2005; Page and Lastra 2003). Thus, it may be suggested that the shift in the stable isotope composition of these long-lived primary consumers is likely an effect of changes not only in the biogeochemistry of primary producers of food items, but also to some extent in the foraging strategies related to alternative regimes. Shifts in diets of these primary producers were nevertheless below 20 % as indicated by gut content analysis, which did not alter their use as planktonic and benthic baselines.

### Shifting from benthic to planktonic energy pathways

While there was variation in species’ trophic niches in the fish communities between clear and turbid water regimes, we found no significant directional food web changes until the community was grouped into trophic guilds, including benthivorous, piscivorous and planktivorous fishes. The trend of decreased benthic reliance and trophic position between the clear water and turbid regimes in time and space was notable in benthivorous and piscivorous fishes, and suggests a shift from benthic to pelagic energy pathways on a trophic basis for the production of these fish species during the regime shift. Additionally, species-specific shifts in trophic position and benthivory due to a regime shift suggest a strong species effect on each trophic guild directing trophic niche shifts, except for planktivorous fishes. Benthivorous and piscivorous fishes shifted in food web space as a result of a regime shift from clear to turbid water, becoming more dependent on plankton-based production. The detected food web shift towards planktonic production was likely due to increasing turbidity being coupled with a shift in local productivity towards the water column. This is known as a loss of benthic energy pathways in response to local environmental change, such as eutrophication (Vadeboncoeur et al. 2003). The loss of this pathway leads to a reduction in ecological efficiency. For example, zoobenthic production has been shown to be more efficiently passed to fish than zooplankton production due to the larger size of benthic relative to planktonic prey (Vander Zanden et al. 2006). Our results showing the decreased trophic position of benthivorous and piscivorous fish species are in line with these suggestions.

### Foraging strategies in alternative regimes

The fish community in Lake Taihu is characterized by a high proportion and richness of omnivorous species. For example, the food items of most species in this study include zooplankton (e.g., crustaceans), zoobenthos (e.g., larvae of insects), planktonic and benthic primary productions (e.g., detritus/algae) (Tables S3 and S4). Although the phytoplankton could be expected to support the majority of the fish community in the turbid regime, planktonic production did not contribute completely to the adult fish populations (e.g., benthic food items were still recorded for benthivorous and piscivorous species). Rather, the energy pathways supporting fish were more benthic in the clear habitat for these fishes, as demonstrated by both stable isotope and dietary evidence. However, the diet content was relatively stable for planktivorous fishes. The zooplanktivorous fish, *N. taihuensis*, only assimilated zooplankton, for example, and omnivorous planktivorous fishes, such as bighead and silver carps, mainly fed on zooplankton and phytoplankton in the planktonic food chain (Tables S3, S4). This relatively conservative feeding strategy can explain why the benthivory and trophic position of these fishes were less sensitive to the regime shift as indicated by the mixing-model results.

Although the increased proportion of planktonic food items in fishes could be a main reason for pelagic energy pathways, another reason for the enhancement of the latter in the turbid water regime could be that the benthic primary consumer prey for fishes actually shifted from benthic to planktonic food items, as discussed above for snails and mussels. In the sediment of our study area, eutrophic tolerant species of zoobenthos, such as *Branchiura sowerbyi* (Oligochaeta: Tubificidae), *Limnodrilus hoffmeisteri* (Oligochaeta: Tubificidae), and *Tanypus chinensis* (Diptera: Chironomidae), dominated the local benthic community (Cai et al. 2010). The planktonic subsidy of these organisms increases along a gradient of phytoplankton abundance (Xu and Xie 2004). Therefore, as eutrophication proceeds, not only zooplankton benefit from the increased phytoplankton production, but many benthic invertebrates can also benefit from increases in the phytoplankton by direct filter feeding or by collecting newly sedimented phytoplankton (Carpenter et al. 1996; Davies 1980; Welch et al. 1988). The increasing subsidy from phytoplankton to benthic secondary consumers could be another important reason for planktonic energy pathways of fish community in turbid regimes.

### Turbid regime as a stable state

In the subtropical shallow lakes, omnivorous/planktivorous fish species, such as lake anchovy, icefish, bighead and silver carps, are dominant and feed both on phytoplankton and zooplankton. Thus, the top-down control of zooplankton by fish is usually strong all year around and the grazing on phytoplankton by zooplankton is low. In addition, the piscivorous fishes are not abundant, facultative piscivores and, as we found, the decreasing trophic position of piscivores during turbid regimes consequently reduced the power of top-down control on secondary consumers. These structural and functional characteristics of trophic interactions do not facilitate piscivore control on these intermediate fishes in the turbid regime (Gelós et al. 2010), and the top-down effect in the classic trophic cascade may thus have little influence on phytoplankton biomass in contrast to bottom-up effects (Jeppesen et al. 2007; Pacheco et al. 2010).

Benthivorous fishes were also found to have high flexibility in foraging strategies under alternative regimes. By intense predation on benthic sediment-dwelling invertebrates these species are known to have an eutrophicating effect, reducing transparency for the piscivorous fishes and facilitating the nutrient release from the sediment, which further enhance the productivity of phytoplankton (Richardson et al. 1995). Meanwhile, since our results supported turbidity-promoted changes in the trophic structure and a shift to reduced benthic energy pathways, the turbid regime would be extremely sensitive to additional nutrient loading because of direct fueling to phytoplankton, making the system more stable towards turbidity (Jeppesen et al. 2010; Scheffer and van Nes 2007). In conclusion, the stability of the turbid regime is achieved by these fish-induced direct and indirect negative effects, which will suppress the system’s response to further environmental changes and restoration efforts, such as a reduction of nutrient loading, and further promotes excessive phytoplankton development (Jeppesen et al. 2007; Scheffer and Carpenter 2003).

## Electronic supplementary material

Below is the link to the electronic supplementary material.

Supplementary material 1 (DOCX 272 kb)
