# Supplementary material for: Contrasting energy pathways at the community level as a consequence of regime shifts
Source: Oecologia. 2014 Jan 12;175(1):231–41. doi: 10.1007/s00442-013-2878-2 (PMC3992223; doi:10.1007/s00442-013-2878-2)
Supplement: Supplementary file 1 — Supplementary material 1 (DOCX 272 kb) [file 442_2013_2878_MOESM1_ESM.docx]

## Electronic Supplementary Material

**Oecologia (MS OECO-D-13-00570)**

**Contrasting** **energy pathways at the community-level as a consequence from regime shifts**

**Jun Xu^1,*^, Zhourui Wen^2^, Zhixin Ke^3^, Meng Zhang^4^, Min Zhang^5^, NiChun Guo ^6^, Lars-Anders Hansson^7^, Ping Xie^1,*^**

**Running Head: regime shift in energy pathways**


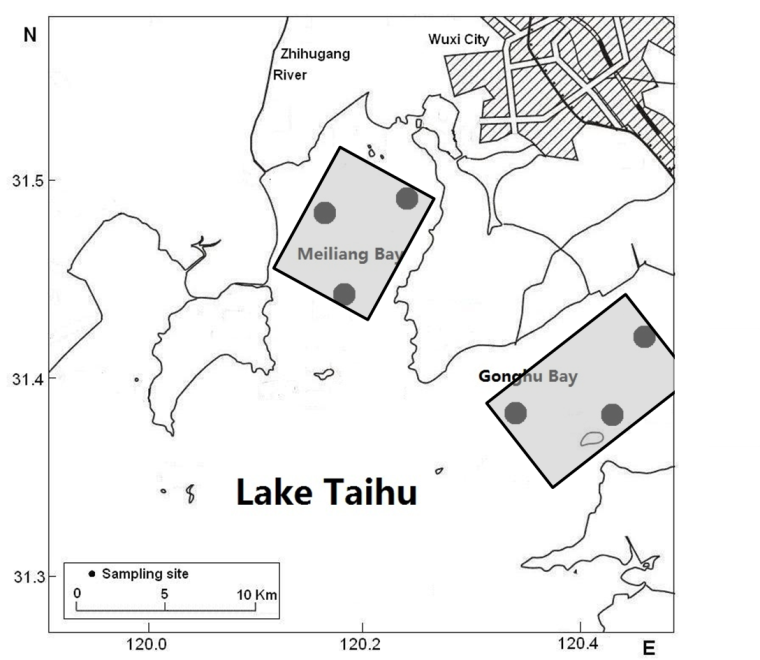


Figure S1. Location of sampling in Lake Taihu. Solid circles represent sampling sites of clear and turbid water regime surrogates and the shaded areas denotes the areas of transects for fishes and baseline organisms sampling.

Table S1. The clear and turbid water regime surrogates (mean ± SD) in each regime. Abbreviations as in Fig. 1-2, and Table 1. Note that these values were averaged by monthly monitoring results from May to October (*n* = 6).

| Regime | Chlorophyll *a* (μg L^-1^) | Total nitrogen (mg L^-1^) | Total phosphorus (mg L^-1^) | Secchi depth (cm) | Submersed macrophyte coverage (%) |
| --- | --- | --- | --- | --- | --- |
| Clear-G | 21.6 ± 6.6 | 1.76 ± 1.2 | 0.11 ± 0.07 | 94.7 ± 9.1 | 63.3^*^ ± 26.7 |
| Turbid-G | 30.8 ± 20.2 | 2.00 ± 0.7 | 0.18 ± 0.03 | 30.0 ± 9.3 | 6.7^**^ ± 2.9 |
| Turbid-M | 52.5 ± 25.2 | 3.01 ± 1.5 | 0.20 ± 0.1 | 25.8 ± 2 | 0 |
| ^*^ The dominant species were *Potamogeton malaianus*, *Hydrilla verticillata*, and *Ceratophyllum demersum*. | | | | | |
| ^**^ The dominant species is *P. malaianus*. | | | | | |

Table S2. Welch two sample *t*-test for differences in mean δ^13^C and δ^15^N of mussels and snails within each regime. Sample size of mussels and snails ranged from 54 to 60. Abbreviations as in Fig 1.

| Regime | δ^13^C | δ^15^N |
| --- | --- | --- |
| Clear-G | *t* = -40.8566, df = 108.875, *P* < 0.0001 | *t* = -24.5032, df = 113.137, *P* < 0.0001 |
| Turbid-G | *t* = -42.46, df = 113.741, *P* < 0.0001 | *t* = -16.9027, df = 111.637, *P* < 0.0001 |
| Turbid-M | *t* = -10.8677, df = 110.345, *P* < 0.0001 | *t* = -17.2523, df = 111.597, *P* < 0.0001 |

Table S3. Dietary data of fish species in alternative regimes presented as proportion (mean ± SD) of each food item. Abbreviations are following Fig. 1-2, and Table 1.

| Trophic guilds | Regime | Species | Fish prey | Zoobenthos | Benthic primary production | Zooplankton | Planktonic primary production | Populations | Number of individuals |
| --- | --- | --- | --- | --- | --- | --- | --- | --- | --- |
| Benthivores | Clear | *Hyporhamphus intermedius* | 0 | 65.5 | 1.8 | 32.7 | 0 | 1 | 5 |
| Benthivores | Clear | *Cyprinus carpio* | 2.3 | 97.7 | 0 | 0 | 0 | 1 | 12 |
| Benthivores | Clear | *Carassius auratus* | 0 | 11 | 65 | 22.4 | 1.6 | 1 | 15 |
| Piscivores | Clear | *Culter erythropterus* | 81.7 ± 5.1 | 17.5 ± 1.6 | 0.4 ± 0.7 | 0.4 ± 0.6 | 0 | 3 | 412 |
| Piscivores | Clear | *Coilia ectenes* | 26.5 ± 33.2 | 73.5 ± 32.3 | 0 | 0 | 0 | 2 | 173 |
| Planktivores | Clear | *Neosalanx taihuensis* | 0 | 0 | 0 | 100 | 0 | 1 | 118 |
| Planktivores | Clear | *Aristichthys nobilis* | 0 | 0 | 10.8 | 74.7 | 14.5 | 1 | 9 |
| Planktivores | Clear | *Hypophthalmichthys molitrix* | 0 | 0 | 6.4 | 59.7 | 33.9 | 1 | 4 |
| Benthivores | Turbid | *Hyporhamphus intermedius* | 0 | 54.1 ± 7.1 | 0 | 45.9 ± 26.7 | 0 | 3 | 321 |
| Benthivores | Turbid | *Cyprinus carpio* | 0 | 70 | 0 | 0 | 30 | 1 | NA |
| Benthivores | Turbid | *Carassius auratus* | 0 | 27.3 ± 4.5 | 45.5 ± 9.2 | 27.3 ± 2.3 | 0 | 2 | 213 |
| Piscivores | Turbid | *Culter erythropterus* | 26.3 | 63.1 | 10.5 | 0 | 0 | 1 | 58 |
| Piscivores | Turbid | *Coilia ectenes* | 3.1 ± 2.4 | 14.2 ± 18.8 | 0 | 82.8 ± 19.3 | 0 | 4 | 981 |
| Planktivores | Turbid | *Neosalanx taihuensis* | 0 | 2.2 ± 1.6 | 0 | 97.8 ± 2.1 | 0 | 4 | 684 |
| Planktivores | Turbid | *Aristichthys nobilis* | 0 | 0 | 0 | 22.1 ± 10.8 | 77.9 ± 10.8 | 2 | 87 |
| Planktivores | Turbid | *Hypophthalmichthys molitrix* | 0 | 0 | 0 | 2.4 ± 2.8 | 97.6 ± 22 | 2 | 67 |
| Note: NA indicates data were not available. | | | | | | | |  |  |

Reference:

Fish laboratory in Institute of Hydrobiollogy, H. p. 1976. Fishes of Yangtze River. - Science Press (in Chinese).

Guo, L. 2005. Studies on fisheries ecology in a large eutrophic shallow lake, Lake Chaohu. - PhD thesis, Chinese Academy of Sciences, China (in Chinese with English abstract).

Liu, E., Bao, C., Wu, L. and Cao, P. 2007. Comparison of food composition and anailsis on mutual effects between *Neosalanx tangkahkeii* Chen and *Taihuensis* Yen et Lin in Lake Taihu. - Journal of Lake Sciences 19: 103-110 (in Chinese with English abstract).

Liu, E., Bao, C. and Yang, Q. 2009. Analysis on the diet content of *Hypophthalmichthys molitrix* (Cuvier et Valenciennes) and *Aristichthys nobilos* (Richardson) under eutrophication in Lake Chaohu. - Journal of Hydroecology 2: 27-31 (in Chinese with English abstract).

Liu, E., Liu, Z., Bao, C. and Wu, L. 2007. Food content and the mutual effect betweent *Hypophthalmichthys molitrix* (Cuvier et Valenciennes) and *Aristichthys nobilos* (Richardson) and *Coilia ectenes taihuensis* Yen et lin in Lake Taihu. - Journal of Lake Sciences 19: 451-456 (in Chinese with English abstract).

Ni, Y. and Zhu, C. 2005. Fishes of the Taihu Lake -Shanghai Scientific and Technical Publishers, China (in Chinese).

Qin, B., Xu, P., Wu, Q., Luo, L. and Zhang, Y. 2007. Environmental issues of Lake Taihu, China. - Hydrobiologia 194: 3-14.

Tang, Y. 1987. On the population dynamics of lake anchovy in Taihu Lake and its rational exploitation. - Journal of Fisheries of China 11: 61-73 (in Chinese with English abstract).

Yang, Q., Liu, E., Bao, C., Wu, C., Wang, S., Li, H. and Song, X. 2008. Analysis of the component of the food for *Neosalanx tangkahkeii taihuensis* in Chaohu Lake. - Journal of Anhui Agricultural Science 36: 15922-15924 (in Chinese with English abstract).

Yang, R., Xie, C. and Yang, X. 2003. Study on the food compasion of six fierce fishes species in Lake Liangzi, China -Reservoir Fisheries 22: 7-8 (in Chinese with English abstract).

Ye, J., Liu, Z. and Wang, W. 2007. Comparative study on the feeding habits of *Coilia ectenes* and *Hyporhamphus intermedius* in the Meiliang Bay of Lake Taihu -Journal of Lake Sciences 19: 218-222 (in Chinese with English abstract).

Zhang, T. 2005. Life-history strategies, trophic patterns and community structure in the fishes of lake Biandantang. - PhD thesis, Chinese Academy of Sciences, China (in Chinese with English abstract).

Table S4 The clear and turbid water regime surrogates in the lakes where the dietary data collected.

| Lake | Regime | Latitude | Longitude | Year | TN (mg/l) | TP (µg/l) | Chla (µg/l) | Secchi (cm) | Macrophyte coverage (%) | References |
| --- | --- | --- | --- | --- | --- | --- | --- | --- | --- | --- |
| Lake Biandantang | Clear | 30°15′ | 114°43′ | 2003–2004 | 1.1 | 32 | 2.3 | 107 | 55 | Zhang (2005) |
| Lake Qingling | Clear | 30°27′ | 114°15′ | 1993–1995 | 1.9 | 25 | NA | NA | 52 | Yu et al. (1996) |
| Lake Liangzi | Clear | 30°15′ | 114°35′ | 1991–1992 | 0.7 | 23 | 12 | 200 | 41 | Jin (1999) |
| Lake Poyang | Clear | 29°01′ | 116°19′ | 1987–1988 | 0.7 | 76 | 2.1 | 80 | 81 | Jiang and Dou (2003); Liu and Zhang (2000) |
| Lake Chaohu | Turbid | 31°29′ | 117°33′ | 2002–2003 | 2.8 | 102 | 40 | 34 | 3 | Guo (2005) |
| Lake Taihu | Turbid | 31°15′ | 120°14′ | 2000 | 2.5 | 100 | 23 | 40 | 5 | Ni and Zhu (2005) |
| Meiliang Bay | Turbid | 31°27′ | 120°12′ | 2005 | 2.5 | 152 | 48 | 31 | <1 | Ke et al. (2012) |

Note: NA indicates characteristics that were not available or not measured for some lakes.

Reference:

Zhang T. 2005. History Strategies, Trophic Patterns and Community Structure in the Fishes of Lake Biandantang. PhD thesis, Chinese Academy of Science.

Ke Z, Xie P, Guo L, Xu J, Zhou Q. 2012. Effects of large bio-manipulation fish pen on community structure of crustacean zooplankton in Meiliang Bay of Taihu Lake. Chinese Journal of applied ecology. 23: 2270-2276.

Liu R, Zhang S. 2000. Multivariable analyzing and comparing of water quality of shallow lakes in middle and lower reaches of Changjiang Reiver. Acta Hydrobiologica Sinica 24: 439-445.

Ni Y, Zhu C. 2005. Fishes of the Taihu Lake: Shanghai Science and Technology of China Press, China.

Jiang J, Dou H. 2003. Five freshwater lakes in China: University of Science and Technology of China Press, China.

Jin G. 1999. State of submersed vegetation resources in lake Liangzi, lake Niushan, and lake Baoan. Acta Hydrobiologica Sinica 23: 87-89.

Guo L. 2005. Study on fisheries ecology in a large eutrophic shallow lake. PhD thesis, Chinese Academy of Sciences, China.
